# Evaluation of Pharmacokinetics of Boronophenylalanine and Its Uptakes in Gastric Cancer

**DOI:** 10.3389/fonc.2022.925671

**Published:** 2022-07-12

**Authors:** Futian Tang, Yujie Wei, Shining Zhang, Jianrong Wang, Wenjiao Gu, Fenxia Tang, Xiaohuan Peng, Yucai Wei, Jiangyan Liu, Weiqiang Chen, Shixu Zhang, Long Gu, Yumin Li

**Affiliations:** ^1^ Key Laboratory of Digestive System Tumor of Gansu Province and Department of Cardiovascular Disease, Lanzhou University Second Hospital, Lanzhou, China; ^2^ South-East Institute of Lanzhou University, Putian, China; ^3^ Nuclear Medicine Department, Lanzhou University Second Hospital, Lanzhou, China; ^4^ Key Laboratory of Heavy Ion Radiation Biology and Medicine, Institute of Modern Physics, Chinese Academy of Sciences, Lanzhou, China; ^5^ School of Nuclear Science and Technology, University of Chinese Academy of Sciences, Beijing, China; ^6^ Department of Radiotherapy Technology, Lanhai Nuclear Medicine Research Center, Putian, China; ^7^ School of Nuclear Science and Technology, Lanzhou University, Lanzhou, China

**Keywords:** boronophenylalanine, boron neutron capture therapy, pharmacokinetics, safety, gastric cancer, MKN45 cells

## Abstract

Boron neutron capture therapy (BNCT), a cellular-level particle radiation therapy, combines boron compounds selectively delivered to tumor tissue with neutron irradiation. Boronophenylalanine (BPA) is a boron compound widely used in malignant melanoma, malignant brain tumors, and recurrent head and neck cancer. However, neither basic nor clinical research was reported for the treatment of gastric cancer using BPA. Selective distribution of boron in tumors rather than that in blood or normal tissue prior to neutron irradiation is required for the successful treatment of BNCT. This study evaluated the pharmacokinetics and safety of ^10^B-labeled BPA (^10^B-BPA, abbreviated as BPA) and its uptakes in gastric cancer. Pharmacokinetics and safety were evaluated in Sprague–Dawley (SD) rats intravenously injected with BPA. The uptakes of boron in gastric cancer cell line MKN45 and in cell-derived xenografts (CDX) and patient-derived xenografts (PDX) animal models were measured. The results showed that the boron concentration in the blood of rats decreased fast in the first 30 min followed by a steady decrease following the observation time, having a half-life of 44.11 ± 8.90 min and an AUC-last of 815.05 ± 62.09 min×μg/ml. The distribution of boron in different tissues (heart, liver, lung, stomach, and small intestine) of rats revealed a similar pattern in blood except for that in the brain, kidney, and bladder. In MKN45 cells, boron concentration increased in a time- and concentration-dependent manner. In both CDX and PDX animal models, the boron is preferentially distributed in tumor tissue rather than in blood or normal tissues. In addition, BPA had no significant adverse effects in rats. Taken together, the results suggested that BPA revealed a fast decrease in boron concentration in rats and is more likely to distribute in tumor cells and tissue.

## Introduction

Boron neutron capture therapy (BNCT) is a radiation therapy at the cellular level and combines neutron irradiation with the boron compounds selectively delivered to tumor tissue (1–3), depending on the nuclear capture and fission reactions (4). These reactions occur when ^10^B, a non-radioactive boron isotope, is irradiated with thermal neutrons to yield α-particles and lithium-7 nuclei with an almost one-cell diameter (5). Therefore, BNCT links two fundamental approaches, i.e., chemotherapy and traditional radiotherapy. L-p-boronophenylalanine (L-BPA) has been clinically used for different kinds of cancers including malignant melanoma (6–8), malignant brain tumors (1, 9, 10), recurrent head and neck cancer (3, 11, 12), and malignant mesothelioma (13, 14). Unlike other radiotherapy, the cytocidal effect of BNCT on tumor tissue relies on the selective uptakes of boron compounds in the tumors. High selectivity of boron in tumor tissue rather than that in blood or normal tissues prior to neutron irradiation is required for the successful treatment of tumors using BNCT (15). In addition, evaluation of boron levels in a tumor is required for dosimetric modeling in BNCT (5, 16). Whole blood concentrations of boron can be used as a surrogate for measuring the boron content in the *in vivo* tissue. Kulvik et al. reported the boron biodistribution after intravenous infusion of BPA-fructose (BPA-F) complex in dogs. They found that the blood boron concentrations were negatively correlated with time after the onset of infusion, while positively correlated with the boron concentrations in the liver, lung, and kidney (16). In addition, Yoshida et al. compared the uptakes of BPA between glioma stem-like cells and their cancerous cells, showing that the boron uptake by the cancerous cells was significantly more than that of glioma stem-like cells (17). In breast cancer cell MCF-7, Hermawan et al. found that the uptakes of boron in the cells increased fast in a time-dependent manner (18). Wang et al. evaluated the biodistribution of ^18^F-BPA in F98 glioma-bearing Fischer 344 rats and found that the concentration of ^18^F-BPA reached the maximum level at 1 h after injection. The tumor/blood ratio showed a steady 2-fold uptake during the 4-h study and the kidneys had the highest radioactivity levels up to 4 h after a single injection (19).

Gastric cancer is a deadly disease with high morbidity and mortality, having poor overall survival statistics throughout the world with 1.06 million new cases in 2020 worldwide (20). It is the fourth and seventh most commonly occurring cancer in men and in women, respectively. However, no basic or clinical research was found to report the effect of BNCT on gastric cancer. The present study was designed to investigate the biodistribution and safety of BPA in rats and the uptakes of boron in gastric cancer cells and tissues, proving the basis and dosimetric modeling for the treatment of gastric cancer using BNCT.

## Materials and Methods

### Animals

The Ethics Committee of Lanzhou University Second Hospital, Lanzhou, China, approved the animal experiment protocol. Eight-week-old male Sprague–Dawley (SD) rats were purchased from the Animal Experimental Center of Lanzhou University, Lanzhou, China. Six-week-old female BALB/c nude mice and 6-week-old NCG mice [gene type: (Prkdc)ko/ko, (IL2rg)ko/ko] were purchased from Gempharmatech, Nanjing, China. The animal experiment was performed according to the *Guide for the Care and Use of Laboratory Animals* of the National Institutes of Health.

### Preparation of BPA-Fructose Complex Solution

**Figure 1:**
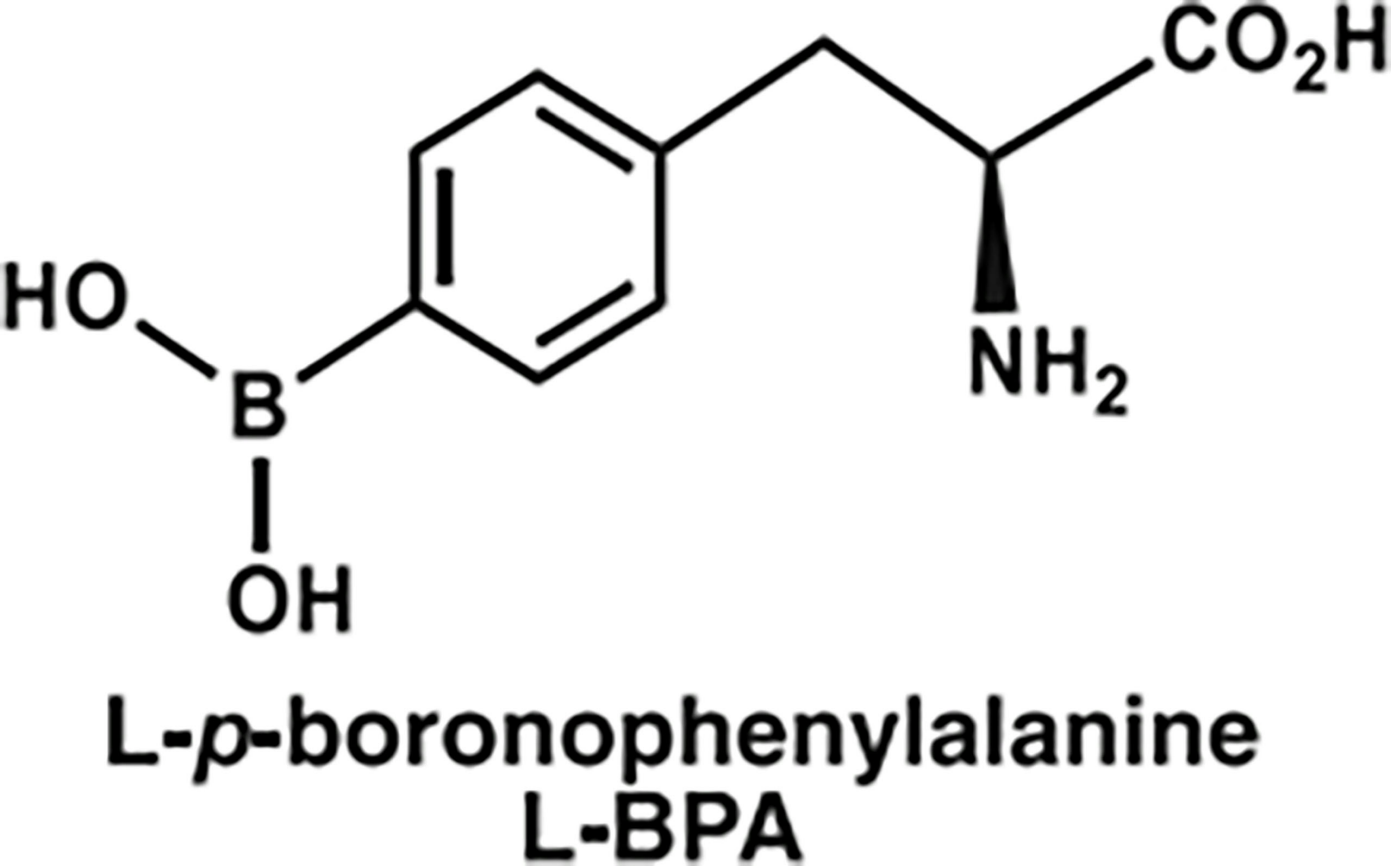
Structure of BPA.

^10^B-labeled BPA (^10^B-BPA, abbreviated as BPA in the text) was provided by Professor Weiqiang Chen (Institute of Modern Physics and Key Laboratory of Heavy Ion Radiation Biology and Medicine, Chinese Academy of Sciences, Lanzhou, China). The structure of BPA is shown in **Figure 1**. Fructose was purchased from Beijing Solarbio Technology Co., Ltd. (Beijing, China). BPA and fructose (at a molar ratio of 1:1.5) were mixed in distilled water followed by adding 1 N NaOH to pH 10.5 (21) under stirring. Then, the pH value was titrated to 7.6 with 1 N HCl. The solution was sterilized by filtrations using a 0.22-μm syringe filter (Merck Millipore, Massachusetts, USA). The final concentration of stock BPA solution was set to 25 mg/ml, having 1.25 mg/ml of boron concentration.

### Preparation of Standard Curve of Boron Concentration

We prepared two series of BPA solutions with final boron concentrations of 0.05, 0.1, 0.2, 0.4, 0.8, and 1.6 μg/ml. Briefly, the stock BPA (25 mg/ml) was diluted 25 times to get working a solution of 1 mg/ml BPA. Different volumes of BPA working solution were added to 100 μl of phosphate buffer solution (PBS) in the first series of experiment, or to 100 μl of rat blood in the second series of experiment followed by the digestion with concentrated acid and measurement of boron concentration. The standard curve of boron concentration was established.

### Measurements of the Boron Concentration

Cellular or tissue samples with or without BPA were digested with a 100-μl 1:1 mixture of concentrated nitric and sulfuric acids for 2 h at 60°C. The digestion solution was diluted by adding 5 ml of 10% nitric acid. The boron concentration in each sample was determined by using inductively coupled plasma atom emission spectroscopy (ICP-AES, PQ9000, Analytik jena GmbH, Jena, Germany) assay.

### Pharmacokinetics of Boron in Rats Intravenously Injected With BPA

After intravenous injection of BPA (125 mg/kg body weight) to six 8-week-old male SD rats, the blood was collected from the eye at 5, 10, 15, 20, 25, 30, 40, 50, 60, 90, 120, 150, and 180 min under anesthesia using isoflurane. One hundred microliters of blood was used for the determination of boron concentration using the ICP-AES assay. The boron concentration was expressed as μg/ml blood. The pharmacokinetics parameters were calculated with the non-compartment model using the Winnonlin software. The curve of boron concentration to time was established.

### Biodistribution of Boron in Rats Intravenously Injected With BPA

At 10, 20, 30, 40, 50, 60, 90, and 120 min after intravenous injection of BPA (125 mg/kg body weight) to 8-week-old male SD rats, 6 animals at each time were anesthetized under isoflurane for collection of blood from the eye. Rats were euthanatized under long-term use of isoflurane for collection of main kinds of tissues, including heart, liver, kidney, lung, brain, glandular stomach, small intestine, and muscle. Boron concentration in 100 μl of blood or 50 mg of tissues was determined using the ICP-AES assay. The boron concentration was expressed as μg/g blood or tissues. Pearson correlation calculation was conducted using all tissues as variables.

### Uptakes of Boron in Gastric Cancer Cell MKN45 Treated With BPA

MKN45 cells originated from gastric adenocarcinoma (Zhili Zhongte Biological Technology Co., Ltd. Wuhan, China) in the exponential growth phase were used to determine the uptake of boron. Gastric adenocarcinoma is the canceration of gastric glandular epithelial cells. We used the MKN45 cell line in both *in vitro* and *in vivo* studies because gastric adenocarcinoma is the main type of gastric cancer, accounting for more than 95% of gastric cancer. The cells were incubated in Petri dishes 10 cm in diameter for 1 and 3 h with BPA (62.5 and 125 ppm expressed as boron). At the end of the incubation, the cells were washed 3 times with cold PBS, harvested with trypsin, and centrifuged at 900 g. The number of cells was counted with Neubauer’s chamber. Boron concentration in cells was measured using ICP-AES and expressed as μg/10^7^ cells.

### Biodistribution of Boron in Normal Tissues or Tumor of Mice Bearing MKN45-Derived Tumors

For the establishment of a cell-derived tumor xenograft (CDX) model, human gastric cancer cells MKN45 (initial 5×10^6^) were subcutaneously injected into 6-week-old BALB/c nude mice. To evaluate the boron uptake, 6 animals were injected intravenously with the BPA at a dose of 250 mg/kg body weight and killed at 1 h after injection. Boron concentration in 100 μl of blood and 50 mg of tumor or normal tissues was measured using ICP-AES and expressed as μg/g tissue.

### Biodistribution of Boron in Normal Tissues or Tumor of Mice Bearing Human Gastric Cancer Tissue

For the establishment of a patient-derived tumor xenograft (PDX) model, fresh gastric cancer specimens in a size of 2–3 mm^3^ were implanted into 6-week-old NCG mice. The 3rd generation of PDX-bearing mice was used for the administration of BPA. Informed written consents were obtained from all patients. The study was approved by the Institutional Ethics Committee of Lanzhou University Second Hospital (permit No. LZUSH-2021-26). To evaluate the boron uptake, 6 animals were injected intravenously with the BPA at a dose of 250 mg/kg body weight and killed at 1 h after administration. Boron concentration in 100 μl of blood and 50 mg of tumor or normal tissues was measured using ICP-AES and expressed as μg/g tissue.

### Safety of BPA in Normal Rats

To verify the safety of BPA in the dose used in the present experiment, we examined the blood cell counts and hemoglobin, blood lipid level, liver function, and kidney function as well as histology of most important tissues in rats at 3 h after intravenous injection of BPA (125 mg/kg body weight) or PBS.

### Statistical Analysis

The data were expressed as the means ± SD. A two-way analysis of variance (ANOVA) was conducted to evaluate the significance of differences. Pharmacokinetics parameters were analyzed with Winnonlin software. The statistical analyses, including Pearson correlation, were evaluated with the SPSS Statistics program (ver. 26, IBM, Endicott, NY). *p* values <0.05 were considered significant.

## Results

### Standard Curve of Boron Concentration

**Figure 2:**
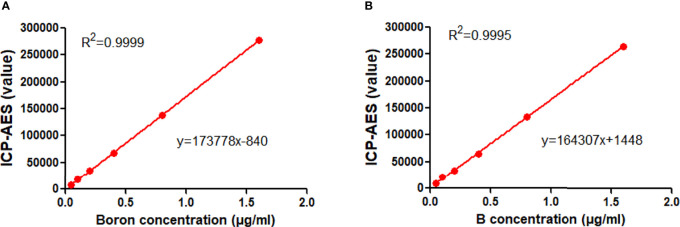
Standard curve of BPA. **(A)** The linear correlation between the ICP-AES value and boron concentrations in the first set of experiment in which BPA was added in PBS. **(B)** The linear correlation between the ICP-AES value and boron concentrations in the second set of experiment in which BPA was added in rat blood.

As shown in **Figure 2A**, for the first series of experiment in which BPA was added to PBS, the linear correlation between ICP-AES value and boron concentrations was found, having a correlation coefficient (*R* ^2^) of 0.9999 and an equation of *y* = 173778*x* − 840. Similarly, the second series of experiment in which BPA was added to rat blood revealed an *R* ^2^ of 0.9995 and an equation of *y* = 164307*x* + 1448 (**Figure 2B**). The first equation was used to calculate the boron concentration in the cells and the second one was used for calculating boron concentration in blood or tissues, including tumors.

### Pharmacokinetics Parameters of Boron in Rats

**Figure 3:**
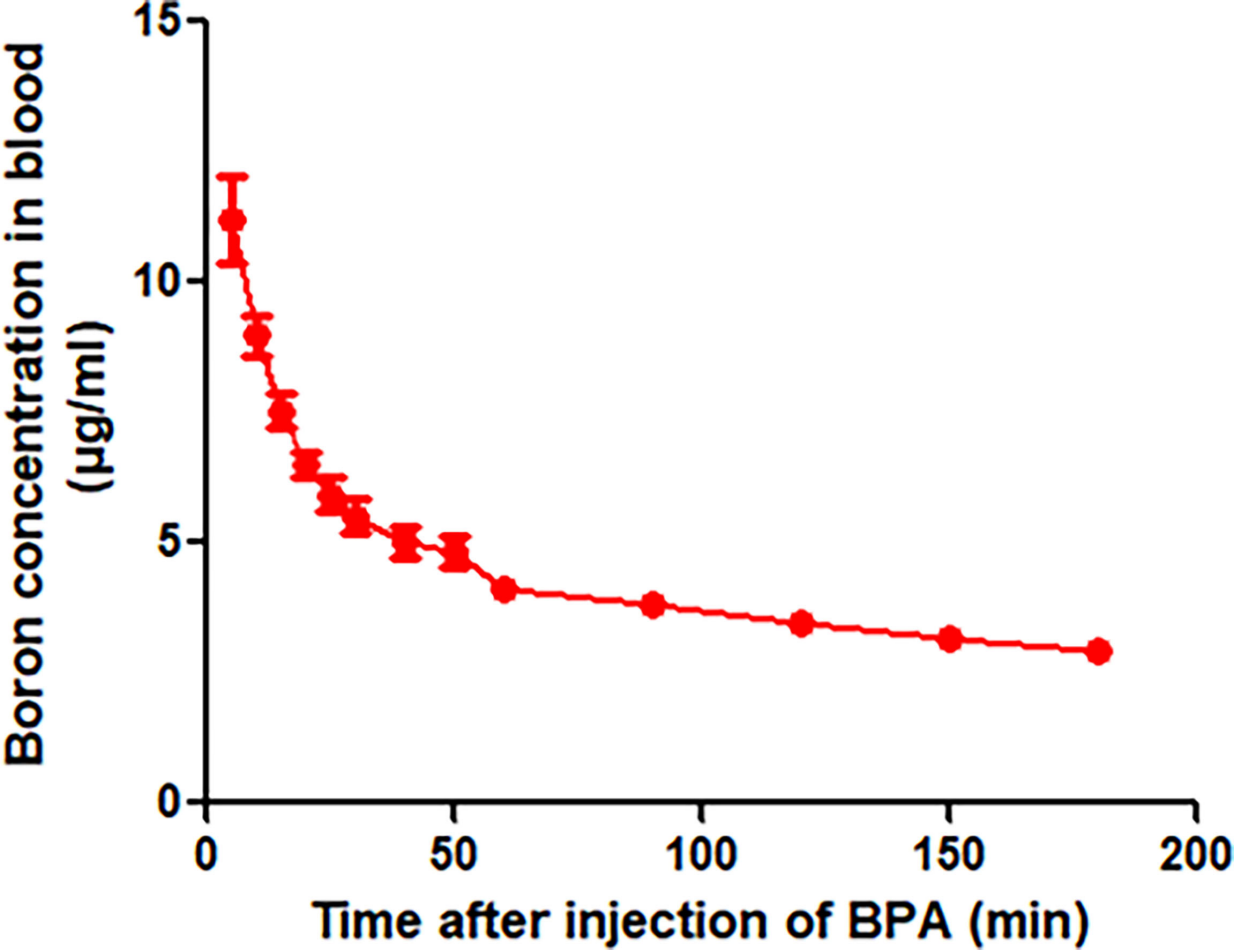
Blood boron concentration–time curve of BPA in rats. Data were expressed as the means ± SD. *N* = 6. The dose of BPA was 125 mg/kg body weight. The age of male rats was 8 weeks. Blood was collected at 5, 10, 15, 20, 25, 30, 40, 50, 60, 90, 120, 150, and 180 min after BPA injection for boron measurement.

To define the protocol of BPA administration in animals, we performed a study on the pharmacokinetics in rats. The analysis of the concentration–time curve showed that the boron concentration in the blood of rats intravenously injected with BPA decreased fast from 11.20 μg/ml in 5 min to 5.51 μg/ml in 30 min (**Figure 3**), followed by a steady decrease from 5.03 μg/ml in 50 min to 2.90 μg/ml in 3 h. As **Table 1** shows, the analysis using a non-compartment model revealed the following boron pharmacokinetics parameters: half-life, 44.11 ± 8.90 min; Tmax, 5.00 ± 0.00 min; Cmax, 11.20 ± 1.66 μg/ml; C0, 14.05 ± 3.18 μg/ml; T-last, 180.00 ± 0.00 min; C-last, 2.90 ± 0.28 μg/ml; AUC-last, 815.05 ± 62.09 min×μg/ml; and AUC-INF, 1,000.10 ± 93.09 min×μg/ml. The results suggested that after intravenous injection of single-dose BPA, the boron concentration decreased quickly in the first 30 min. Therefore, the following experiments were designed based on the features of this pharmacokinetics.

**Table 1:** Pharmacokinetics of BPA in rats.

Items	Unit	Mean ± SD
**Half-life**	min	44.11 ± 8.90
**Tmax**	min	5.00 ± 0.00
**Cmax**	μg/ml	11.20 ± 1.66
**C0**	μg/ml	14.05 ± 3.18
**T-last**	min	180.00 ± 0.00
**C-last**	μg/ml	2.90 ± 0.28
**AUC-last**	min×μg/ml	815.05 ± 62.09
**AUC-INF**	min×μg/ml	1,000.10 ± 93.09

Data are expressed as the means ± SD. Tmax, the time at maximal concentration; Cmax, the maximal concentration; C0, the concentration at 0 min; T-last, the time at the last point; C-last, the concentration at the last point; AUC-last, the area under the curve at the last point; AUC-INF, the area under the curve at the infinitive point.

### Boron Distribution in Normal Tissues of Rats

**Figure 4:**
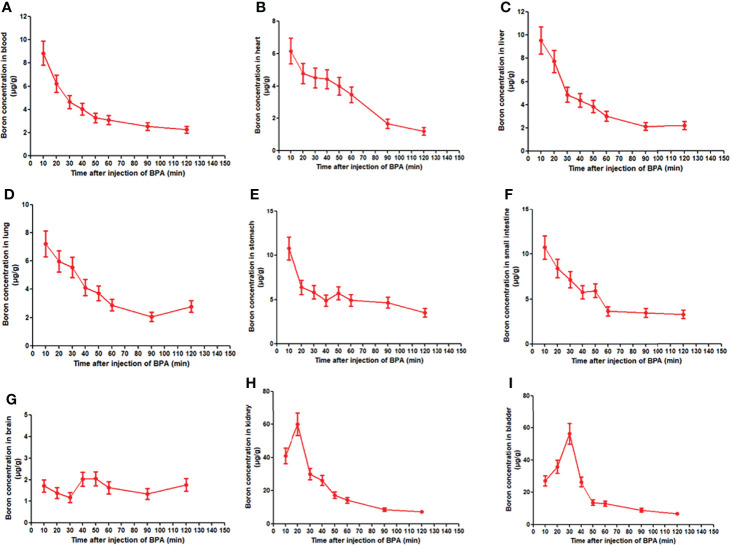
Boron concentration–time curve of BPA in blood **(A)** and heart **(B)**, liver **(C)**, lung **(D)**, stomach **(E)**, small intestine **(F)**, brain **(G)**, kidney **(H)**, and bladder **(I)** of rats. Data were expressed as the means ± SD. *N* = 6. The dose of BPA was 125 mg/kg body weight. The age of male rats was 8 weeks. Blood and tissues were collected at 10, 20, 30, 40, 50, 60, 90, and 120 min after BPA injection for boron measurement.

Given that the boron concentration in the blood of rats showed a fast decrease following a single dose administration of BPA, we investigated boron distribution in normal tissues of rats. The analysis of the concentration–time curve showed that the boron concentration in blood decreased in a time-dependent manner (**Figure 4A**), which were consistent with the findings in the pharmacokinetics study as aforementioned. Similar patterns of boron concentration–time changes were found in the heart (**Figure 4B**), liver (**Figure 4C**), lung (**Figure 4D**), stomach (**Figure 4E**), and small intestine (**Figure 4F**) except that the boron concentration in the brain showed a relatively steady state during the observation (**Figure 4G**). However, the boron concentration in the kidney increased to 60.11 μg/g in 20 min from 40.90 μg/g in 10 min, followed by a fast decrease during the observation (**Figure 4H**). In addition, the boron concentration in the bladder continuously increased to 56.27 μg/g in 30 min from 27.23 μg/g in 10 min, followed by a fast decrease during the observation (**Figure 4I**). To elaborate on this finding, a Pearson correlation calculation was conducted using all tissues as variables (**Table 2**). Blood boron concentrations showed significantly positive correlation with the boron concentrations in heart (*r* = 0.866, p < 0.01), liver (*r* = 0.986, *p* < 0.01), lung (*r* = 0.945, *p* < 0.01), stomach (*r* = 0.943, *p* < 0.01), intestine (*r* = 0.966, *p* < 0.01), and kidney (*r* = 0.813, *p* < 0.05). Brain boron concentrations were not correlated with that in any other tissues. Taken together, these results suggested that the fast decrease of boron concentration in blood might be attributed to the fast excretion through the kidney and bladder. These results also indicated that no boron accumulation was found in the tissues after a single dose of BPA.

**Table 2:** Correlation analysis of boron concentrations in the analyzed samples in rats.

	Blood	Heart	Liver	Lung	Brain	Stomach	Intestine	Kidney	Bladder
**Blood**	1								
**Heart**	0.866^**^	1							
**Liver**	0.986^**^	0.878^**^	1						
**Lung**	0.945^**^	0.891^**^	0.957^**^	1					
**Brain**	−0.111	0.067	−0.085	−0.151	1				
**Stomach**	0.943^**^	0.816^*^	0.900^**^	0.840^**^	−0.028	1			
**Intestine**	0.966^**^	0.904^**^	0.975^**^	0.977^**^	−0.084	0.903^**^	1		
**Kidney**	0.813^*^	0.771^*^	0.884^**^	0.860^**^	−0.0250	0.614	0.843^**^	1	
**Bladder**	0.189	0.636	0.535	0.718^*^	−0.497	0.350	0.622	0.657	1

^*^p < 0.05; ^**^p < 0.01.

### Boron Uptakes in MKN45 Cells

**Figure 5:**
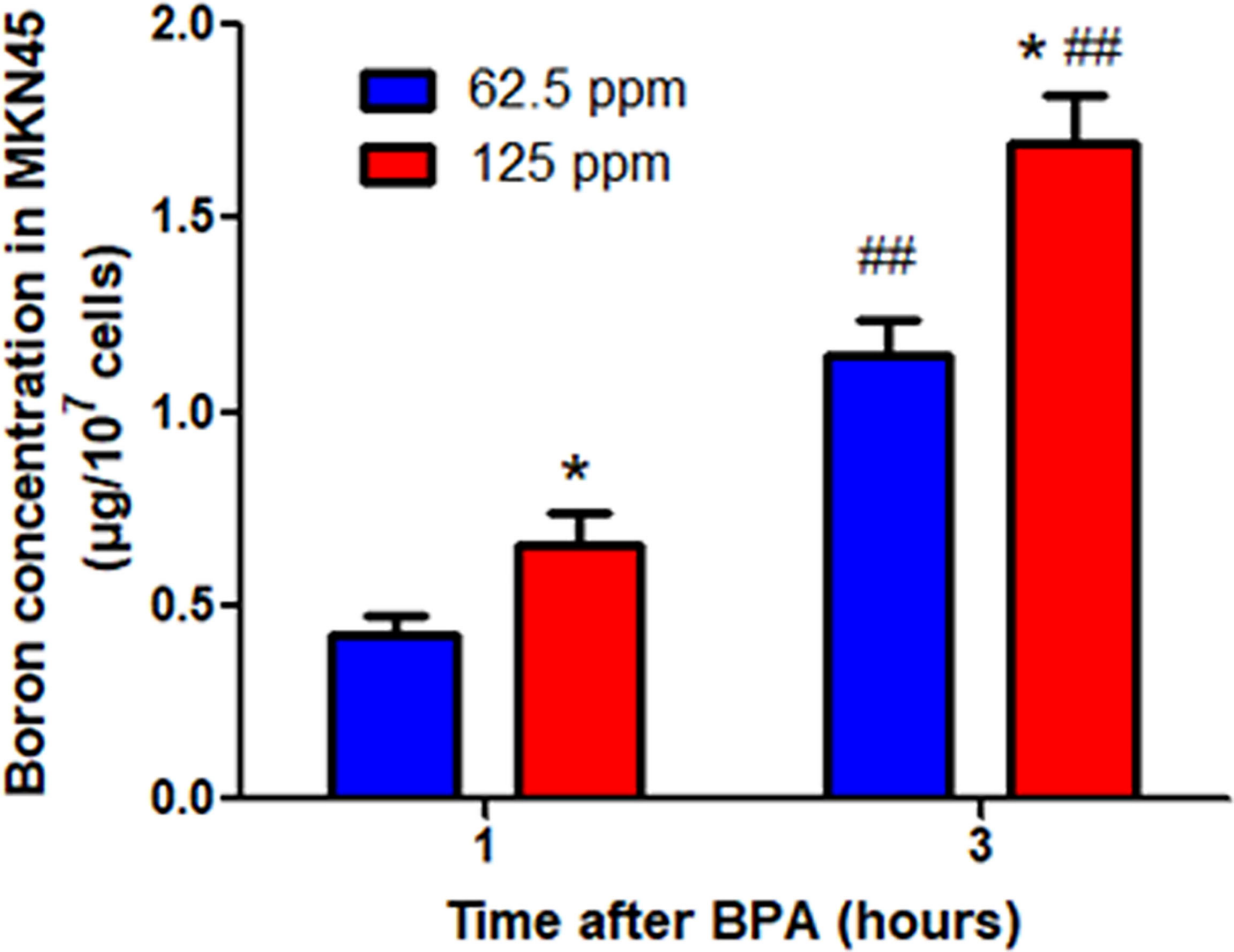
Boron concentration in MKN45 cells. Data were expressed as the means ± SD. *N* = 4. ^*^ *p* < 0.05 compared at the same time; ^##^ *p* < 0.01 compared at the same concentration.

BNCT is a new approach for therapy of cancer, and the first requirement for BNCT is the effective uptake of boron. To test whether MKN45, the gastric cancer cell line, efficiently uptakes the boron, we incubated cells with different concentrations of BPA at the different times. The results demonstrated that the boron concentrations in cells (1.15 μg/10^7^ cells and 1.70 μg/10^7^ cells) at 3 h were higher than that (0.42 μg/10^7^ cells and 0.66 μg/10^7^ cells) at 1 h, respectively (**Figure 5**). The result suggested that MKN45 cells uptook the boron in a time- and concentration-dependent manner, providing the basis for the treatment of gastric cancer using BNCT.

### Boron Distribution in Tumor or Normal Tissue of Mice Bearing MKN45-Derived Tumors

**Figure 6:**
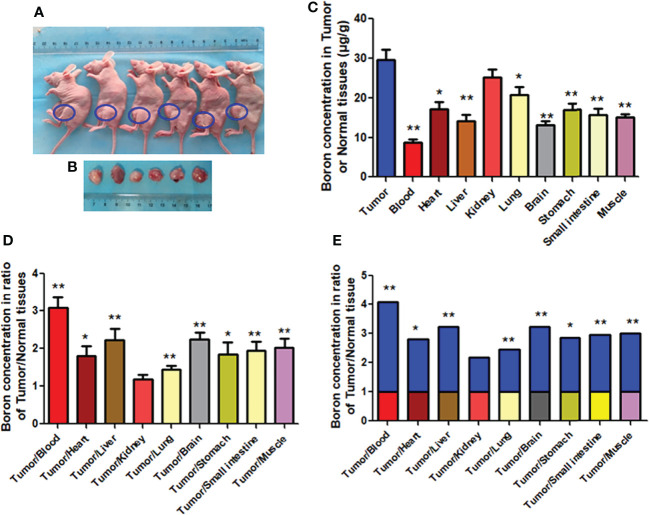
Boron concentration in gastric tumor or tissues of CDX mice. **(A**, **B)** Mice bearing tumor. **(C)** The boron concentration in tumor, blood, and tissues of heart, liver, kidney, lung, brain, stomach, small intestine, and muscle. **(D**, **E)** Boron concentration ratios in tumor/blood or tumor/tissue. Data were expressed as the means ± SD. *N* = 6. The dose of BPA was 250 mg/kg body weight. The age of female mice was 8 weeks. Blood and tissues were collected at 1 h after BPA injection for boron measurement. ^*^ *p* < 0.05, ^**^ *p* < 0.01 compared with tumor or tumor/tissue.

This *in vivo* study examined whether boron preferentially distributes in tumor tissue in CDX mice bearing MKN45 cells rather than in blood or normal tissues. As shown in **Figures 6A, B**, mice bearing tumors were used. The boron concentration in tumor (29.58 μg/g) was significantly higher than that in blood (8.81 μg/g), heart (17.14 μg/g), liver (14.17 μg/g), lung (20.75 μg/g), brain (13.27 μg/g), stomach (17.03 μg/g), small intestine (15.71 μg/g), and muscle (15.02 μg/g) (**Figure 6C**), having boron concentration ratios in tumor/blood or tumor/tissue of 3.09, 1.81, 2.22, 1.45, 2.24, 1.85, 1.95, and 2.01, respectively (**Figure 6D**). Same results were expressed in **Figure 6E** as fold changes of boron concentration in tumor to that in other tissues. However, boron concentration in the tumor was comparable with that in the kidney, having a boron concentration ratio in tumor/kidney of 1.19 (**Figures 6D, E**). These results suggested that the distribution of boron was more likely to be in tumor tissue rather than in blood or normal tissues.

### Boron Distribution in Tumor or Normal Tissue of Mice Bearing Human Gastric Cancer Tissue

**Figure 7:**
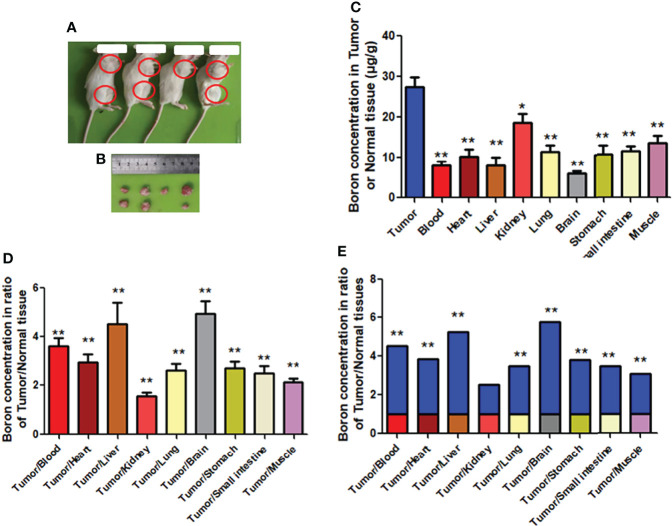
Boron concentration in gastric tumor or tissue of PDX mice. **(A**, **B)** Mice bearing tumor. **(C)** The boron concentration in tumor, blood, and tissues of heart, liver, kidney, lung, brain, stomach, small intestine, and muscle. **(D**, **E)** Boron concentration ratios in tumor/blood or tumor/tissue. Data were expressed as the means ± SD. *N* = 6. The dose of BPA was 250 mg/kg body weight. The age of female mice was 12 weeks. Blood and tissues were collected at 1 h after BPA injection for boron measurement. ^*^ *p* < 0.05, ^**^ *p* < 0.01 compared with tumor or tumor/tissue.

To further confirm the preferential distribution of boron in tumor tissue in the CDX model, we used mice bearing tumor originated from human gastric cancer tissue, namely, the PDX model. Similar to findings from the CDX model, results of the PDX model (**Figures 7A, B**) revealed very significantly higher boron concentration in tumor (27.40 μg/g) than in blood (8.01 μg/g), heart (10.30 μg/g), liver (8.09 μg/g), lung (11.51 μg/g), brain (5.96 μg/g), stomach (10.57 μg/g), small intestine (11.40 μg/g), and muscle (13.46 μg/g) (**Figure 6C**), having boron concentration ratios in tumor/blood or tumor/tissue of 3.61, 2.93, 4.49, 2.62, 4.94, 2.69, 2.50, and 2.13 respectively (**Figure 7D**). The same results are expressed in **Figure 7E** as fold changes of boron concentration in tumor to that in other tissues. Unlikely, boron concentration in tumor was slightly but significantly higher than that in the kidney, having a boron concentration ratio of tumor/kidney of 1.57 (**Figures 7D, E**). Consistent with the CDX model, the present study suggested that boron is preferentially distributed in tumor tissue rather than in blood or normal tissues.

### Safety Evaluation of BPA in Rats

**Figure 8:**
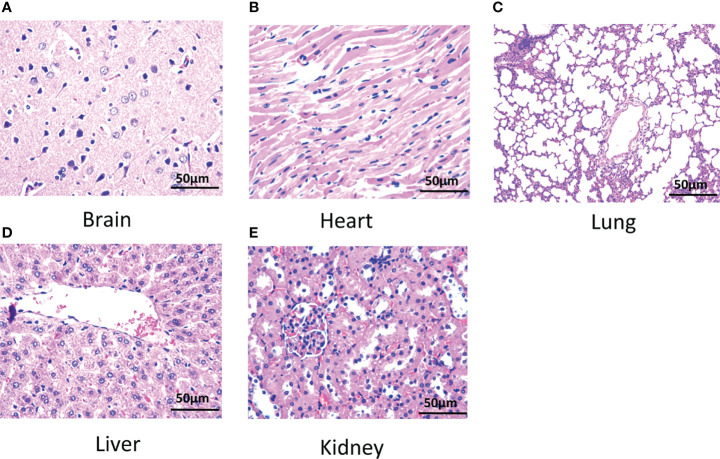
Histology of tissues of rats. **(A)** Brain, **(B)** heart, **(C)** lung, **(D)** liver, and **(E)** kidney. *N* = 6. The dose of BPA was 125 mg/kg body weight. The age of male rats was 8 weeks. Blood and tissues were collected at 3 h after BPA injection for safety evaluation.

The results showed that the cell counts including white blood cells and red blood cells of rats intravenously injected with BPA were comparable with that in rats injected with PBS shown in **Table 3**. Similarly, BPA administration did not affect levels of blood glucose, blood lipid concentration including total cholesterol and triglyceride, liver function including total bilirubin, alanine aminotransferase, and aspartate aminotransferase, as well as kidney function including urea, creatinine, and uric acid (**Table 4**). Histological results revealed no abnormalities in the structure of the brain (**Figure 8A**), heart (**Figure 8B**), lung (**Figure 8C**), liver (**Figure 8D**), and kidney (**Figure 8E**).

**Table 3:** Effect of BPA on the blood cell counts in rats.

Items	Unit	Control	BPA	*p*
**White blood cell**	10^9^/L	10.74 ± 2.70	12.89 ± 3.99	0.406
**Neutrophil**	10^9^/L	6.22 ± 2.15	7.51 ± 1.16	0.334
**Lymphocyte**	10^9^/L	6.20 ± 1.85	5.32 ± 1.74	0.515
**Monocytes**	10^9^/L	0.47 ± 0.22	0.54 ± 0.47	0.791
**Eosinophil**	10^9^/L	0.31 ± 0.08	0.27 ± 0.07	0.528
**Red blood cell**	10^12^/L	8.13 ± 0.52	7.65 ± 0.69	0.308
**Hemoglobin**	g/L	163.75 ± 7.41	163.00 ± 15.79	0.934
**Platelet**	10^9^/L	839.75 ± 243.14	958.75 ± 344.38	0.593

Data are expressed as the means ± SD. N = 6. The dose of BPA is 125 mg/kg body weight. The age of male rats is 8 weeks. Blood was collected 3 h after BPA injection for cell counts.

**Table 4:** Effect of BPA on the biochemical parameters in rats.

Items	Unit	Control	BPA	*p*
**Glucose**	mmol/L	5.94 ± 0.76	8.99 ± 2.07	0.033
**Lipid in blood**				
**Total cholesterol**	mmol/L	1.50 ± 0.13	1.38 ± 0.19	0.362
**Triglyceride**	mmol/L	1.74 ± 0.12	1.66 ± 0.21	0.534
**Liver function**				
**Total bilirubin**	μmol/L	2.68 ± 0.76	2.53 ± 0.57	0.763
**Direct bilirubin**	μmol/L	1.95 ± 0.93	1.55 ± 0.40	0.458
**Indirect bilirubin**	μmol/L	1.78 ± 0.39	1.58 ± 0.42	0.509
**Alanine aminotransferase** **(ALT)**	U/L	52.50 ± 5.92	48.75 ± 8.50	0.496
**Aspartate aminotransferase** **(AST)**	U/L	145.29 ± 36.89	136.00 ± 43.20	0.756
**AST/ALT**		2.74 ± 0.43	2.82 ± 0.78	0.864
**Kidney function**				
**Urea**	mmol/L	6.96 ± 1.80	7.29 ± 1.70	0.803
**Creatinine (CR)**	μmol/L	40.23 ± 6.63	39.08 ± 3.74	0.773
**Uric acid**	μmol/L	102.68 ± 21.93	98.98 ± 18.80	0.806
**Urea/CR**		0.19 ± 0.08	0.18 ± 0.05	0.959

Data are expressed as the means ± SD. N = 6. The dose of BPA is 125 mg/kg body weight. The age of male rats is 8 weeks. Blood was collected 3 h after BPA injection for measurement of biochemical parameters.

## Discussion

We found in the present study that the boron concentration in the blood of rats decreased fast in the first 30 min followed by a steady decrease throughout the experiment, having a very short half-life and less AUC after a single dose of BPA intravenously injected. In MKN45 cells, boron concentration increased in a time- and concentration-dependent manner. In both CDX and PDX animal models, boron is preferentially distributed in tumor tissue rather than in blood or normal tissues. In addition, BPA had no significant adverse effects in rats.

BNCT is an innovative form of radiotherapy, combining neutron irradiation with the boron compounds selectively delivered to tumor tissue (22). Therefore, the first requirement for BNCT is the preferential and effective uptake of boron compounds in tumor cells. BPA has been clinically used for BNCT in the treatment of malignant melanoma (6, 8), malignant brain tumors (10, 23), recurrent head and neck cancer (11, 12, 24), and malignant mesothelioma (13, 14). However, no literature was found to report the treatment of BPA for gastric cancer.

We firstly performed the study on the pharmacokinetics in rats intravenously injected with BPA in order to define the protocol of BPA administration in animals. The results revealed that the boron concentration in blood decreased in a time-dependent manner throughout the experiment after a single-dose injection, having a half-life of 44.11 min. The results are consistent with the previous reports showing that blood boron concentration of mice administered with BPA *via* the tail vein was time-dependently decreased after the injection of boron (17). In the clinical situation, Fukuda et al. reported a half-life of 48 min after infusion of BPA to human patients (6, 25). These results implied that multiple doses or continuous infusions of BPA should be recommended to maintain the high and steady boron concentration in blood, subsequently pushing enough boron into tumor tissue.

The biodistribution curve of boron in blood or normal tissues in rats showed that boron concentration in blood and other tissues decreased in a time-dependent manner except that in the brain, kidney, and bladder. A Pearson correlation calculation revealed that boron concentrations in blood were positively correlated with the boron concentrations in the heart, liver, lung, stomach, intestine, and kidney. Brain boron concentrations were not correlated with that in any other tissues. Consistently, Kulvik et al. reported that blood boron concentrations after the end of the infusion showed a descending pattern with time (16). They also found that boron concentrations in tissues varied greatly, but with the exception of brain and kidney tissue (16). Taken together, these results suggested that the fast decrease of boron concentration in blood might be attributed to the fast excretion from the kidney and bladder.

Gastric cancer is a deadly disease with poor overall survival statistics (20). BNCT might be a new approach for therapy of gastric cancer (4). The results on MKN45 cells, the gastric cancer cell line, incubated with BPA demonstrated that the boron concentrations in cells increased in a time- and concentration-dependent manner. This is the first time to report the preferential uptakes of boron in gastric cancer cells. Consistently, in breast cancer cells, Hermawan et al. reported a high boron concentration in MCF-7 and MDA-MB 231 cells (18). In glioma cells, Yoshida et al. found that the boron uptake by the cancerous cells was significantly higher than that of normal cells (17).

In the clinical situation, boron concentration ratios of tumor/blood or tumor/normal tissue should be more than 2.5 times for effective treatment using BNCT. The study on BPA-injected CDX model mice bearing MKN45 cells showed that boron concentration in the tumor at 1 h after BPA injection was significantly higher than that in blood and other important tissues, including heart, liver, and lung, having a tumor/blood ratio of more than 3 times or tumor/tissues ratios of approximately 2 times, respectively. These results suggested that the distribution of boron was more likely to be in tumor tissue rather than in blood or normal tissues. Similarly, in a glioma-bearing rat model, Wang et al. reported that the uptake of BPA in F98 glioma reached a maximum at 1 h after the drugs’ administration, indicating that 1 h after BPA injection would be the optimal time for BNCT (19). In the clinical situation, Fukuda et al. reported that in patients with melanoma treated with BPA, the melanoma/blood ratio of boron ranged from 2.1 to 3.8 and the skin/blood ratio of boron was 1.31-0.22 (6).

CDX models are widely used to determine the antitumor effects of drug candidates. However, CDX models cannot recapitulate complex human cancer components such as the tumor microenvironment and the heterogeneity (26–28). PDX, in which tumor samples from patients were implanted into immunodeficient mice, have therefore become a favored preclinical model for investigating tumor biology (28–30). The results on BPA-injected PDX model mice revealed a significantly higher boron concentration in tumors than in blood and tissues. Consistent with the CDX model, the present study suggested that the distribution of boron is preferentially distributed in tumor tissue rather than in blood or normal tissues. In the patient with melanoma or glioblastoma treated with BPA, Fukuda found that the half-life of boron concentration was 0.7–3.7 h (25), and the tumor/blood ratio of boron concentration ranged from 1.4 to 4.7 for glioblastoma and was 3.40 for melanoma (25).

BPA itself was reported to be safe when administrated to animal or human (4). The present study on safety showed that BPA in a dose of 125 mg/kg injected into rats for 3 h had no adverse effects on blood cell counts, blood glucose, blood lipid levels, liver function, and kidney function. Histological results revealed no abnormality in structure of the brain, heart, lung, liver, and kidney.

This study has several limitations. First, the study is a preliminary investigation on the biodistribution of BPA in normal rats using only one dose of BPA at different times. Second, in CDX and PDX tumor models, only one dose of BPA at one time was used for evaluating the biodistribution in normal or tumor tissues. Third, only early responses but not later responses were analyzed when the safety of BPA was evaluated. Fourth, a neutron irradiation experiment for therapy of gastric cancer and for safety of BPA was not conducted. These limitations will be solved in a future study.

In conclusion, the results suggested that BPA revealed a fast decrease in boron concentration in rats and was more likely to distribute in tumor cells and tissue.

## Data Availability Statement

The original contributions presented in the study are included in the article/supplementary material. Further inquiries can be directed to the corresponding authors.

## Ethics Statement

The studies involving human participants were reviewed and approved by Institutional Ethics Committee of Lanzhou University Second Hospital. The patients/participants provided their written informed consent to participate in this study. The animal study was reviewed and approved by The Committee on the Ethics of Animal Experiments of Lanzhou University Second Hospital.

## Author Contributions

FTT, YJW, SNZ, JW, WG, and FXT conducted the experiments. FTT, JL, and WC wrote the manuscript. SXZ and LG analyzed the data. XP, YCW, LG and YL conceived the work and revised the manuscript. All authors contributed to the article and approved the submitted version.

## Funding

The study was supported by the Boron Neutron Capture Tumor Treatment Device (AB-BNCT) Project Entrusted by Enterprises and Institutions (20206200050009T2), the National Natural Science Foundation of China (81960673 and 81870329), the Natural Science Foundation of Gansu Province (21JR1RA135), the Cuiying Technological Innovation Foundation of Lanzhou University Second Hospital (CY2019-MS03), the Industrial Support Program for Colleges and Universities in Gansu Province (2020C-04), and the Special Research Project of Lanzhou University Serving the Economic and Social Development of Gansu Province (054000282). Foundation of Gansu Province (No. 20JR5RA321, 2020B-034) and Lanzhou University Second Hospital (CY2019-QN07).

## Conflict of Interest

The authors declare that the research was conducted in the absence of any commercial or financial relationships that could be construed as a potential conflict of interest.

## Publisher’s Note

All claims expressed in this article are solely those of the authors and do not necessarily represent those of their affiliated organizations, or those of the publisher, the editors and the reviewers. Any product that may be evaluated in this article, or claim that may be made by its manufacturer, is not guaranteed or endorsed by the publisher.
